# CD36 overexpression: a possible etiopathogenic mechanism of atherosclerosis in patients with prediabetes and diabetes

**DOI:** 10.1186/s13098-017-0253-x

**Published:** 2017-07-18

**Authors:** M. D. Lopez-Carmona, M. C. Plaza-Seron, A. Vargas-Candela, F. J. Tinahones, R. Gomez-Huelgas, M. R. Bernal-Lopez

**Affiliations:** 1 Internal Medicine Department, Biomedical Institute of Malaga (IBIMA), Regional University Hospital of Malaga (Carlos Haya Hospital), Avda. Hospital Civil s/n, 29009 Malaga, Spain; 2 Research Laboratory-Allergy Unit, Biomedical Institute of Malaga (IBIMA), Regional University Hospital of Malaga (Carlos Haya Hospital), Malaga, Spain; 3 Endocrinology and Nutrition Department, Biomedical Institute of Malaga (IBIMA), Regional University Hospital of Malaga (Virgen de la Victoria Hospital), Malaga, Spain; 4 CIBERFisiopatología de la Obesidad y Nutrición, Instituto de Salud Carlos III, Madrid, Spain

**Keywords:** Atherosclerosis, Type 2 diabetes, CD36 receptor, Human clinical, Monocytes

## Abstract

**Rationale:**

CD36 is a scavenger receptor located on monocytes which is involved in foam cell transformation.

**Aim:**

To evaluate CD36 expression under different glycemic states in both healthy subjects and in atherosclerotic patients.

**Subjects and methods:**

In order to evaluate the possible effects of hyperglycemia on CD36 expression in healthy subjects, an in vitro experiment was carried out using monocyte in three different conditions: extreme hyperglycemia (HG), euglycemia (EG) and in the absence of glucose. On the other hand, three groups of atherosclerotic patients were evaluated according to their glycemic conditions: normoglycemic (NG), prediabetic (preDM) and diabetic (DM) patients. CD36 expression (mRNA, non-glycated and glycated protein) was analyzed in monocytes.

**Results:**

CD36 mRNA expression in the in vitro experiment peaked at 4 and 24 h under HG conditions. No differences in mRNA levels were found in the EG and control group. The level of non-glycated proteins was higher in HG and EG conditions compared with control group. Glycated protein expression was inhibited by glucose in a sustained manner. In atherosclerotic patients, a significant association was observed when comparing glycated CD36 protein expression in DM with NG patients (p = 0.03). No significant differences were found in mRNA and non-glycated CD36 expression in these patients. Moreover, BMI, insulin, weight and treatment were shown to be related to CD36 expression (mRNA, non-glycated and glycated protein levels, depending of the case) in atherosclerotic patients.

**Conclusions:**

Hyperglycemia is an important modulator of CD36 mRNA and non-glycated protein expression in vitro, increasing de novo synthesis in healthy subjects. In atherosclerotic patients, there are progressive increases in CD36 receptors, which may be due to a post-translational stimulus.

## Background

Cardiovascular diseases are very prevalent pathologies in the general population. They affect the majority of adults over 60 years of age and are the main cause of death in developed countries. Although efforts have been made in recent years to reduce this cause of death, the rate of reduction has slowed in recent decades [1, 2]. Epidemiological studies have identified different cardiovascular risk factors such as age, gender, obesity, family history of cardiovascular disease, smoking habits, dyslipidemia, hypertension and diabetes mellitus (DM).

In diabetic patients, atherosclerosis is the most frequent cardiovascular disease. It develops at a young age and in an extensive manner. In fact, premature vascular disease is the main cause of morbidity and mortality among diabetic patients [3]. Hyperinsulinemia can promote monocytes to produce foam cells and contribute to the development of atherosclerosis in diabetic patients [4]. Moreover, the increased prevalence of atherosclerotic disease is not explained by the presence of other cardiovascular risk factors in prediabetic (preDM) patients [5].

The pathophysiological mechanisms involved in the increased risk of cardiovascular disease are not entirely clear. The development of atherosclerotic damage has been attributed to lipoprotein accumulation in the intima layer, especially an accumulation of low-density lipoproteins (LDL) [6]. After crossing the endothelium, these lipoproteins are modified [7, 8] and taken up by macrophages and smooth muscle cells [9] through two principal mechanisms: by specific LDL receptors (LDL-R) [10, 11] and by scavenger receptors [12].

CD36 is a scavenger receptor with a high affinity for oxidized LDL (oxLDL) [13]. It is expressed by various types of cells such as platelets, macrophages, adipocytes and smooth muscle cells. It binds oxLDL with a high affinity and, in monocytes–macrophages, CD36 is responsible for the transformation into foam cells in 60–70% of cases [14, 15]. This receptor is synthesized as non-glycated protein (55 KD). It then undergoes various cytoplasmatic post-translational changes to transform it into a glycated protein (88 KD) that is anchored to the cellular surface. CD36 has been described as having a pathogenic effect during atherosclerotic vascular disease due the direct relationship between this pathology and the expression of the receptor. In fact, overexpression of this receptor could be one of the mechanisms behind accelerated atherosclerosis in diabetic patients [16]. However, some evidence has shown that this expression could be affected by a change in hyperglycemic conditions [17]. Moreover, recent studies have shown that higher levels of this marker in the blood can be used as potential marker of cardiovascular disease [18, 19].

The aim of this study was to analyze CD36 expression in peripheral blood monocytes in healthy subjects in different cell culture conditions: extreme hyperglycemia (HG), euglycemia (EG) or without glucose (saline solution) (control group). Furthermore, we evaluated whether different CD36 expression levels were associated with different degrees of glucose metabolism alteration in normoglycemic (NG), prediabetic (preDM) and diabetic (DM) patients.

## Patients and methods

This experiment consisted of two parts. The first part was a preliminary in vitro study with the objective of analyzing the influence of hyperglycemia on CD36 receptor expression. The second part was a study carried out in patients diagnosed with severe atherosclerosis, in which we measured the CD36 expression in different states of carbohydrate metabolism.

### In vitro experiments in healthy subjects

Peripheral blood mononuclear cells (PBMC) were isolated from three bags of red blood cell concentrate from healthy donors with AB Rh+ blood type. Cells were separated by standard density gradient centrifugation with Ficoll (Sigma-Aldrich, St. Louis, MO, USA) (400*g*, for 40 min, at room temperature (22 °C) without stopping). One million monocytes per well were grown over 48 h in monolayer cultures on plates using Gibco^®^ RPMI 1640 cell culture medium (Gibco, Invitrogen Co., Carlsbad, CA, USA) supplemented with glutamine.

To compare the effects of hyperglycemia on CD36 mRNA, non-glycated and glycated protein expression, PBMC were exposed to different glucose (Sigma-Aldrich) concentrations (5.5, 26 mM or without glucose in a 0.9% saline solution), to simulate euglycemic (EG), hyperglycemic (HG) or control conditions, respectively. These glucose concentrations were determined in relation to normal plasma glycemic levels (5.5 mM = 95 mg/dL) and in relation to very high plasma glycemic levels, in the postprandial situation (26 mM = 498 mg/dL). Samples of cell cultures were harvested at time 0 and at 0.5, 1, 2, 4, 8, 16, 24 and 48 h to measure CD36 expression. Results were obtained by three independent experiments performed in duplicate. The cells were conserved in TriPure Isolation Reagent (Roche Molecular Biochemicals, Mannheim, Germany) at −80 °C until the time of RNA and protein isolation. From cultures which were collected at 8, 16 and 48 h, protein phases were separated to determinate glycated CD36 and non-glycated CD36.

### Experiments in atherosclerotic patients

A total of 32 patients with documented advanced cardiovascular disease (coronary, cerebrovascular or peripheral) were included in this study. The clinical-epidemiological data of their clinical histories was taken into account. They came from University Regional Hospital from Malaga and were treated surgically by Cardiovascular Surgery Service in 2007 and 2008.

Atherosclerotic patients were subclassified into three groups: patients with type 2 diabetes mellitus (DM), patients with pre-diabetes (preDM) and normoglycemic patients (NG). DM patients were defined as individuals who follow oral antidiabetic treatment and/or insulin treatment and have HbA1c levels over 6.5%. Subjects without DM were considered preDM if they had HbA1c levels in a range of 5.7–6.4% and NG if this level was lower than 5.7% [3].

Patients with severe associated diseases, those in a terminal phase, those with severe mental illness, alcoholism or drug addiction, those under age 18 or over 80, those with hematological diseases or congenital or acquired immunodeficiency syndrome and those who had been treated with immunosuppressant or corticoid drugs during the prior year were excluded.

Clinical and epidemiological data and prescribed treatment at the time of inclusion in the study were obtained from clinical histories. The following parameters were taken into account:

Cardiovascular risk factors including gender, age, diabetes, HTN and tobacco use. Quantitative variables including weight, BMI, total cholesterol, LDL, HDL, triglycerides, glucose, HOMA-IR and HbA1c levels. Treatment with the follow drugs was also considered: antihypertensives, lipid-lowering drugs, oral antidiabetics, insulin and antiaggregants.

The classification of diabetes patients was carried out using the *ADA 2010* [3] definition according to HbA1c levels, as previously described. In accordance with the Spanish Society of Cardiology’s Guidelines on Arterial Hypertension criteria [20], hypertensive individuals were defined as those whose systolic arterial pressure was ≥140 mmHg and/or whose diastolic arterial pressure was ≥90 mmHg, measured on two separate occasions separated by at least two weeks. NCEP-ATPIII 2001 [21] criteria were used in determining hypercholesterolemia. Hypercholesterolemia was defined as values of total cholesterol ≥200 and LDL ≥160 mg/dL on repeated occasions. Healthy lipid control criteria were defined as an optimal LDL cholesterol level ≤100 mg/dL [21]. Statin use prior to the diagnosis of a cardiac event was also a factor in determining if a subject had hypercholesterolemia. In terms of HDL levels, limits were ≤40 mg/dL in men and ≤50 mg/dL in woman. Hypertriglyceridemia was defined as triglycerides levels ≥150 mg/dL on repeated occasions or being under specific treatment at the time of inclusion [21]. Individuals were considered as obese if they had a body mass index (BMI) above 30 kg/m^2^. Patient reporting was used to determine if an individual was a smoker. To be considered an ex-smoker, the patient must have stopped their smoking habit at least 6 months prior to inclusion in the study.

After considering the influence of hyperglycemia on CD36 expression, those patients with fasting glucose levels ≥126 mg/dL during sample collection were also excluded. The final sample size included 22 subjects.

Peripheral blood samples were obtained after subjects fasted and did not take medication for at least 12 h prior to the phlebotomy. These samples were used to measure biochemical levels and for cell culture. Blood was collected in trisodium citrate (3.8%) for use in biochemical testing and in EDTA tubes for cell culture studies. PBMCs were isolated by density gradient centrifugation with Ficoll, as described above, and they were selected according their diameter using a Coulter counter. They were then frozen in TriPure Isolation Reagent (−80 °C) until use.

### CD36 receptor expression analysis

#### CD36 mRNA isolation and quantification

Isolation of CD36 mRNA was performed with TriPure Isolation Reagent (Roche Molecular Biochemicals) following the manufacturer’s protocol. RNA purification was carried out using a commercial kit (Qiagen). RNA purity quality was determined according to the 260/280 ratio using a Thermo Scientific NanoDrop 2000. Samples with ratios from 1.7 to 2 were considered suitable for expression studies. 2 µg/µL of total RNA was used for reverse transcription to cDNA using a High Capacity cDNA Archive Kit (Applied Biosystems, Carlsbad, CA, USA) in a GeneAmp^®^ PCR System 9700 Thermal Cycler, following the manufacturer’s instructions. CD36 cDNA relative quantitation and real-time PCR with the ∆∆C_t_ method was performed using TaqMan probes (Hs00169627_m1) with 18S rRNA as an endogenous control gene (Hs99999901-s1). PCRs were carried out with 2 µL of cDNA, 25 µL of PCR Master Mix (PEBiosystem, Carlsbad, CA, USA) and 2 µL of TaqMan probes according to the follow schedule: 95 °C, 10 min (DNA polymerase activation) and 40 cycles at 95 °C for 15 s followed by 60 °C for 1 min, in ABI PRISM 7900 Detection System (Applied Biosystems).

#### Glycated CD36 and non-glycated CD36 protein isolation and quantification

Total proteins were isolated using TriPure Isolation Reagent. They were then precipitated with absolute ethanol and washed with guanidine hydrochloride. They were quantified using a BCA Protein Assay Reagent. Protein sample concentration was standardized in order to carry out CD36 quantification using a Western Blot, following the protocol previously described [22]. Total protein separation was carried out under denaturing conditions. Transfer was done using nitrocellulose membranes, commercial iBlot Gel Transfer Stacks and an iBlot Dry Blotting System (IB 1001, Invitrogen) at 23 V for 7 min. Then, blots were incubated with monoclonal antibodies against human CD36 (ab17044; Abcam; dilution 1:400). Equal protein loading in each line was verified staining filters with Ponceau and also by incubating blots with monoclonal antibodies against B-actin (clone AC-15, Sigma). ChemiDoc (Bio-Rad, Mississauga, ON, Canada) was used to quantify western blot bands using Quantity One 1-D Analysis Software. Results were expressed as arbitrary units (AU) defined as units of intensity/mm^2^.

### Statistical analysis

CD36 mRNA expression was used for sample size calculation. Thus, based on a standard deviation of 14.41 (mean value of 51.28) our sample size was calculated to have more than 80% power to detect a difference of approximately 40% in mRNA expression at α = 0.05. Following this statistical approach the minimal sample size required was 7 subjects.

The confidence interval was calculated at 95%. Differences were considered significant if p < 0.05. Results were expressed as mean ± SD. Clinical characteristics of patients were compared using the Mann–Whitney U test. Statistical significance between different groups was calculated using ANOVA. Qualitative variables were compared using Chi square test. The relationship between CD36 and continuous variables was analyzed using the Spearman rank correlation, in which the statistical index to measure a lineal relationship between two quantitative variables was (−1 < r < 1). To correct and identify the possible confounding factors in CD36 expression, such as cardiovascular risk factors or drugs, a logistic regression model was used.

Statistical analyses were calculated using SPSS Statistics 20.0 for Windows (IBM Corporation Inc., Somers, NY, USA).

## Results

### In vitro experiment

#### Effects of hyperglycemia on mRNA synthesis

The results of CD36 mRNA in healthy subjects (Table 1) showed that in the absence of glucose (saline solution) (control group), CD36 expression was stable during the experiment. In cell cultures with NG, there was an increase in mRNA synthesis that measured 2.3 times higher (0.5 h) than the control group. This difference, however, was not statistically significant. Statistically significant differences between CD36 mRNA in both groups were not observed at other points. However, in HG cell cultures, an increase of CD36 mRNA expression (1.8 times higher than the control group) was detected at 0.5 h, though it was not statistically significant. This was followed by statistically-significant increases at 4 and 24 h, with measurements that were 18.1 and 5.4 times higher, respectively, when compared with the control group.

**Table 1:** CD36 data at the different time points analyzed in this study

Glucose concentration	Incubation time								
	0 h	0.5 h	1 h	2 h	4 h	8 h	16 h	24 h	48 h
Normalized CD36 (CD36 mRNA expression/18s rRNA)									
Control: saline (0 mg/dL)	1.80	1.86	1.84	1.67	1.81	1.79	1.89	2.90	4.00
EU conditions: 5.5 mM (95 mg/dL)	1.80	4.14	0.76	1.08	1.01	2.05	3.06	4.40	7.39
HG conditions: 26 mM (498 mg/dL)	1.80	3.28	0.96	1.60	32.8	3.57	1.78	15.6	4.19
Arbitrary non-glycated CD36 protein concentration levels									
Control: saline (0 mg/dL)	–	–	–	–	–	7.70	8.95	–	6.37
EU conditions: 5.5 mM (95 mg/dL)	–	–	–	–	–	7.20	8.31	–	13.39
HG conditions: 26 mM (498 mg/dL)	–	–	–	–	–	6.33	1.92	–	6.65
Arbitrary glycated CD36 protein concentration levels									
Control: saline (0 mg/dL)	–	–	–	–	–	14.70	12.43	–	13.99
EU conditions: 5.5 mM (95 mg/dL)	–	–	–	–	–	10.20	11.89	–	5.59
HG conditions: 26 mM (498 mg/dL)	–	–	–	–	–	3.77	3.14	–	3.17

#### Effects of hyperglycemia on non-glycated CD36 synthesis

Table 1 shows non-glycated CD36 protein expression at 8, 16 and 48 h. At 8 h, non-glycated CD36 concentration was similar in all groups. However, at 16 h a maximum peak of protein synthesis was detected in the HG culture, which was 1.6 times higher than that of the control group. At 48 h, CD36 expression in HG returned to basal levels. On the other hand, in the EG cell culture an increase in non-glycated CD36 protein concentration was measured at 48 h that was 2.1 times higher than that of the control group. In the control group, levels remained stable over the course of the entire experiment.

#### Effects of hyperglycemia on glycated CD36 synthesis

Table 1 summarizes glycated CD36 concentration at 8, 16 and 48 h. Glycated CD36 concentrations in the control group were stable over the course of the 48 h. In EG cell culture, lower levels of glycated CD36 concentration were measured in comparison with control group. These levels remained stable until the end of the experiment, although at 48 h, a decrease in CD36 concentration (2.5 times lower than the control group) was detected, which was even lower than the base levels. Glycated CD36 concentration in the HG culture was inhibited during the entire period of the experiment in comparison with the control group.

### Results of the experiment on atherosclerotic patients

#### Patient characteristics

**Fig. 1:**
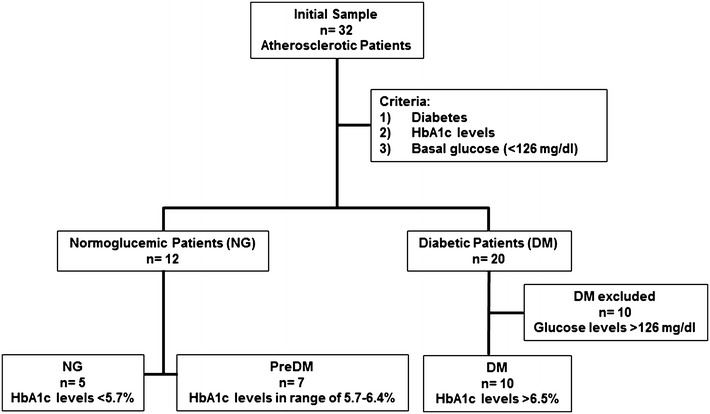
Selection algorithm for the different groups of atherosclerotic patients

22 atherosclerotic patients were included: 10 DM, 7 preDM and 5 NG (Fig. 1). Table 2 summarizes anthropometric and analytical variables of the 3 groups of atherosclerotic patients. A high prevalence of cardiovascular risk factors was observed in our population. Of 22 patients, 14 had a diagnosis of coronary disease, 3 had significant obstruction of the carotid arteries and 5 had peripheral artery disease. No significant differences were found among the groups in the clinical parameters analyzed with the exception of HbA1c levels (p = 0.02). It is important to note that no significant differences were found in fasting blood glucose levels among the three groups. All levels measured were considered NG.

**Table 2:** Anthropometric and analytic variables and treatment of the atherosclerotic patients groups

Variable	NG	Pre DM	DM	p value
Anthropometric and analytical variables				
N	5	7	10	0.4
Age (years)	59 ± 11	66 ± 14	61 ± 8	0.5
Weight (Kg)	76.1 ± 13.7	71.8 ± 13.4	91.6 ± 24.8	0.1
BMI (Kg/m^2^)	27.5 ± 3.8	26.3 ± 4.1	33 ± 3.4	0.2
SBP (mm Hg)	131 ± 21	139 ± 18	121 ± 23	0.3
DBP (mm Hg)	73 ± 15	81 ± 16	68 ± 9	0.2
Glucose (mg/dL)	94 ± 5	97 ± 11	101 ± 11	0.4
Total cholesterol (mg/dL)	153 ± 53	158 ± 38	138 ± 47	0.7
LDL-C (mg/dL)	96 ± 40	106 ± 27	71 ± 30	0.1
HDL-C (mg/dL)	32 ± 7	28 ± 10	41 ± 22	0.3
Triglycerides (mg/dL)	119 (95–189)	135 (112–170)	122 (100–183)	1.0
HbA1c (%)	5.3 ± 0.3	5.9 ± 0.2	6.5 ± 1.0	*0.02*
Insulin (µU/mL)	14.2 ± 2.5	11.3 ± 6.0	13.2 ± 7.6	0.8
HOMA index	3.3 ± 0.6	2.8 ± 1.7	3.3 ± 2.1	0.9
Cardiovascular risk factors				
Gender (% males)	80	57	80	0.5
Arterial hypertension (%)	80	57	100	0.1
Dyslipidemia (%)	80	57	60	0.7
Smoking habits (%)	60	29	10	0.1
Ex-smokers	40	14	20	
Non-smokers	0	57	70	
Obesity (%)	60	29	70	0.2
Overweight	20	43	20	
Normal weight	20	28	10	
Treatment N (%)				
Oral antidiabetics (ADO)	0 (0%)	0 (0%)	8 (80%)	
Metformin	0 (0%)	0 (0%)	7 (70%)	
Sulfonylureas	0 (0%)	0 (0%)	3 (30%)	
Insulin	0 (0%)	0 (0%)	4 (40%)	
Lipid-lowering drugs	2 (40%)	3 (42.9%)	8 (80%)	0.1
Statins	2 (40%)	3 (42.9%)	8 (80%)	0.1
Antihypertensives	4 (80%)	4 (57.1%)	10 (100%)	0.1
ACE inhibitors	2 (40%)	1 (14.3%)	4 (40%)	0.4
ARBs	0 (0%)	1 (14.3%)	5 (50%)	0.1
Beta blockers	1 (20%)	3 (42.9%)	4 (40%)	0.4
Antiaggregants	3 (60%)	5 (71.4%)	9 (90%)	0.2
ASA	2 (40%)	4 (57.1%)	9 (90%)	0.1
Clopidogrel	1 (20%)	1 (14.3%)	0 (0%)	1.0

Data shown as mean ± SD

**Fig. 2:**
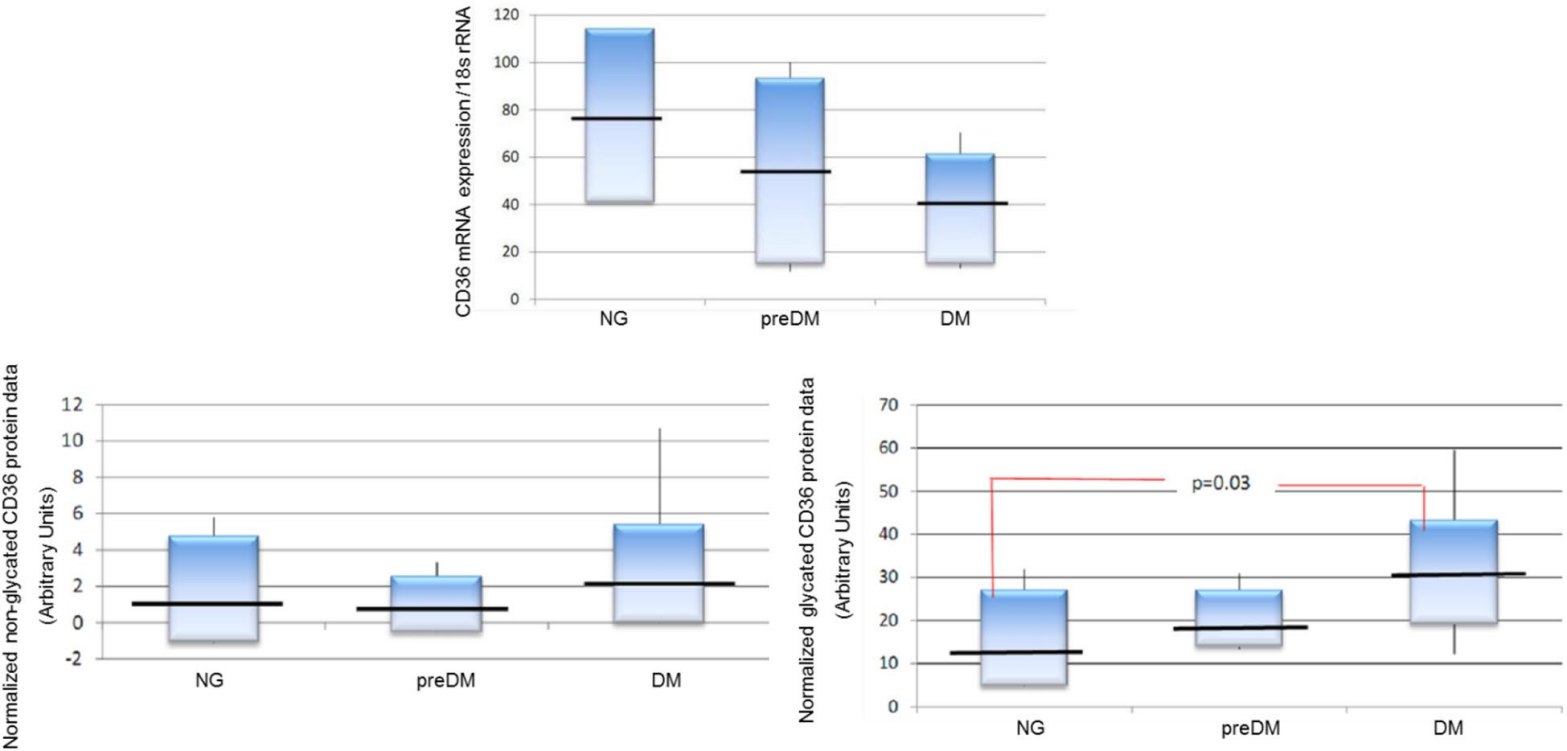
CD36 expression results in atherosclerotic patients. Results are expressed as mean ± SD. mRNA CD36: normalized CD36 mRNA data (CD36 mRNA expression/18s rRNA). Non-glycated CD36: normalized non-glycated CD36 protein data (in arbitrary units); Glycated CD36: normalized glycated CD36 protein data (in arbitrary units); NG: normoglycemic; PreDM: prediabetic; DM: diabetic patients

*CD36 expression (mRNA and non*-*glycated and glycated protein levels) in atherosclerotic patients* Results of CD36 mRNA in atherosclerotic patients are shown in Fig. 2. mRNA synthesis was higher than in NG patients compared to preDM patients and the level was higher in preDM patients compared to DM patients, although these differences were not statistically significant. Lower levels of non-glycated CD36 protein were found in atherosclerotic patients, though there were no statistically significant differences between the groups (Fig. 2).

Glycated CD36 expression showed a proportional increase that was associated with the level of glucose alteration present in patients. In this sense, in NG patients the mean was 15.9 ± 8.9 units, in preDM patients it was 20.4 ± 6.9 units and in DM patients it was 31.1 ± 16.7 (Fig. 2). Although a statistically significant trend was not observed between the three groups, a significant association was observed when in glycated CD36 protein expression in DM compared to NG patients (p = 0.03).

*Multiple regression between CD36 expression (mRNA and non*-*glycated and glycated protein levels) and clinical factors* Multiple regression analysis was performed to analyze whether CD36 expression was related to the presence of the main clinical factors (gender, age, diabetes, arterial hypertension and smoking habits). Taking glycated CD36 as a dependent variable, the results showed that CD36 expression was related to only diabetes, which was related with glycated CD36 (β = 0.46, p = 0.03). However, in multiple regression analyses which included quantitative variables such as weight, BMI, total cholesterol, LDL, HDL, triglycerides, glucose, insulin, HOMA-IR, and HbA1c, the follow relationships were observed: CD36 mRNA levels were associated with insulin levels (β = −0.59, p = 0.02); non-glycated CD36 was associated with BMI (β = 0.72, p = 0.001) and glycated CD36 was associated with weight (β = 0.71, p = 0.001).

All treatments (antihypertensives, lipid-lowering drugs, oral antidiabetics, insulin and antiaggregants) were taken into account when conducting the analyses, but only metformin was related with glycated and non-glycated CD36 protein expression [(β = 0.47, p = 0.03), (β = 0.61, p = 0.003) respectively)].

## Discussion

Atherosclerosis has become one of the most important public health issues in recent decades. This pathology is especially severe among diabetic patients. With the objective of analyzing the influence of glucose metabolism on the atherogenic process, our study focused on the scavenger receptor CD36 in peripheral cells (monocytes), which is a key element in the formation of atheromas. We have found that hyperglycemic conditions have been shown to be responsible for the increase in CD36 (mRNA and non-glycated protein) synthesis in healthy subjects. However, this condition can inhibit glycated CD36 protein expression, as has been found in patients with poorly-controlled diabetes in another study carried out by our group [17]. On the other hand, in atherosclerotic patients, progressive (both, mRNA and glycated) CD36 overexpression has been detected in preDM y DM individuals and is attributed to a post-translational stimulus related with the hyperglycemic state of these patients, a concept which has been previously proposed by other authors [23].

Hyperglycemia has been described as an important stimulator of in vitro CD36 expression, both on the mRNA [24] and protein levels [23–26]. Our results are in agreement with some of these studies which have indicated that hyperglycemia is a stimulus of mRNA synthesis. This effect seems to fluctuate as time passes (Fig. 1), which would explain the different results obtained by other authors [24, 27]. In our study, it has been detected that glucose can influence the de novo synthesis of non-glycated CD36 proteins, which appeared sooner and in higher levels when the glucose concentration was higher; this effect, however, seemed to be limited by time. Higher glucose concentration also inhibited glycated CD36 expression in healthy subjects, as has been reported [27], though other authors have found contrary results [23, 25]. Inhibition was shown since the beginning of the study and was not as important in monocytes cultured in lower glucose levels (EG conditions), which later showed a decrease in glycated CD36 protein expression. These results indicate that expression inhibition is directly related with glucose concentration. The results of this study have shown that hyperglycemia is a powerful inhibitor of glycated CD36. These results are in agreement with other studies previously published by our group [17]. As such, in order to avoid interference in the analyses carried out in our study, we eliminated all individuals whose illness was poorly controlled, defined as those who had glucose levels that were higher than 126 mg/dL at the time of blood extraction from the study, before starting with the experiments.

The impact of acute hyperglycemia on atherosclerosis is not clear. Among the effects described are plasma lipoprotein alterations, an increase in free radicals and an increase in leukocyte adhesion molecules as a consequence of the increase in blood glucose levels. Some of these factors, such as IL-4 or oxLDL, are responsible for regulation of CD36 expression [28, 29]. In our study, atherosclerotic patients were not shown to have statistically significant differences in mRNA levels among the different groups, but results indicated an inverse relationship between glucose metabolism alteration and CD36 mRNA synthesis (Fig. 2). Moreover, differences in non-glycated CD36 levels between the patients groups were not observed (Fig. 2). Although it has been stated that glucose can regulate CD36 protein/mRNA? expression at the translational level [23], in our study, lower non-glycated protein levels indicated that mRNA translation to proteins was regulated independently from glucose metabolism alteration. Furthermore, atherosclerotic patients showed differences in glycosylated CD36 protein expression in DM, preDM and NG states, with statistically significant differences observed when comparing DM and NG patients (Fig. 2). This indicates that alterations in glucose metabolism can increase glycosylated CD36 protein expression by increasing post-translational modifications. Currently, there is controversy regarding the influence of glucose control in diabetic patients and CD36 mRNA/glycated/non-glycated?? expression [30]. It is thought that the difference in patients’ degrees of atherosclerosis could explain the differences in the results. Moreover, glucose control could affect CD36 expression in poorly-controlled atherosclerotic subjects, inducing increased expression of CD36 in these patients [16]. On the contrary, in diabetic patients without atherosclerotic disease, it was found that CD36 mRNA/glycated/non-glycated?? expression rates were higher in well-controlled patients [30]. In our study, all patients’ glucose levels were very well-controlled, with a mean HbA1c of 6.5 ± 1.0%. As such, we hypothesize that additional mechanisms could play a role. Taking into account our results (mRNA, non-glycated and glycated proteins in NG, preDM and DM patients), we believe that in diabetic patients, there could be a higher concentration of CD36 in cytoplasmic granules that would be quickly mobilized under hyperglycemic conditions, as has been proposed by other authors [30]. In this sense, our results suggest that higher levels of CD36 mRNA/glycated/non-glycated?? expression are due to an increase in recycling, because non-glycated CD36 was stable for the duration of the study in the three patient groups, mRNA levels were lower in NG patients compared with preDM patients and both groups had lower mRNA levels with respect to DM patients. Nevertheless, we cannot discount the possibility of other factors such as increased efficiency in post-translational changes, especially glycosylation, as a response to glucose metabolism alteration, as has been propose by other authors.

Current type 2 diabetes diagnostic criteria include a cutpoint beyond which hyperglycemia is associated with the development of microvascular lesions, especially retinopathy [3]. However, atherosclerotic macrovascular disease occurs in the prediabetic stages [31]. In our study, we have analyzed preDM patients with an overexpression of CD36 as compared with NG and we hypothesize that this mechanism could be involved in the early atherogenesis observed in prediabetic stages.

CD36 is a key element in the relationship between atherosclerosis and glucose metabolism, as it has multiple ligands and it is present in some tissues where this receptor acts as mediator in this process. On the one hand, CD36 mRNA/glycated/non-glycated?? expression has been related to atherosclerotic development in the general and the diabetic population [30, 32, 33]. CD36 levels correlate with development of diabetic microvascular complications, especially nephropathy [34, 35]. On the other hand, it has been demonstrated that soluble CD36 in plasma is an insulin-resistance marker [33, 36]. At the adipocyte level, oxLDL uptake by the receptor causes the appearance of insulin-resistance [37] by means of the interaction of multiple CD36 ligands with the serine/threonine kinase system that regulates the ligand-dependent signaling of the insulin receptor [38]. Moreover, CD36 has been related with the inflammatory response present in obesity and metabolic syndrome [39]. However, it is not clear if the higher CD36 mRNA/glycated/non-glycated?? expression found in DM patients is exclusively due to insulin resistance. Published data are contradictory on the effect of CD36 on the development of insulin resistance. Some studies have been carried out on segments of the Japanese population with a congenital CD36 deficit which have shown that this deficit could be both a risk factor and a protective factor in the development of insulin resistance [40, 41] and it has been suggested that this discordance could be due to the presence of pathological molecule development, such as oxLDL in a pro-inflammatory environment [42]. Insulin has the capacity to act on immune cells, increasing CD36 mRNA/glycated/non-glycated? expression and increasing the capacity for oxLDL molecule uptake [43]; in our results, an inverse relationship with CD36 mRNA levels has been observed.

Other authors have postulated that CD36 mRNA/glycated/non-glycated?? overexpression in the insulin-resistance stages could be related to certain characteristics of obesity. Along these lines, we have found a relationship between both glycated CD36 expression and weight and non-glycated CD36 and BMI. This data could indicate that obesity would be a post-translational stimulus for CD36 mRNA/glycated/non-glycated?? expression. In another study carried out by our group, it was found that levels of glycated CD36 were significantly higher in patients with 3 or 4 atherosclerotic risk factors than in patients with a lower number of risk factors in subjects with no atherosclerotic symptoms [17]. That study described the importance of weight as an atherosclerotic risk factor.

CD36 expression in patients in this study has also been shown to be related with the use of metformin. This drug increased glycated and non-glycated CD36 expression [(β = 0.47, p = 0.03) and (β = 0.61, p = 0.003), respectively]. Our results differ from previously published data, where metformin has been shown to have the capacity to decrease in vitro CD36 mRNA/glycated/non-glycated?? expression in skeletal muscle cells [44, 45] and macrophages, [46] but not in adipocytes [47].

In our study, an increase of CD36 mRNA and non-glycated expression in HG conditions in healthy subjects has been observed. This is a process which oscillates over time. However, upon analysis, differences exist in glycated proteins, which are inhibited by HG conditions. On the other hand, glycated CD36 expression is higher in DM patients compare to preDM. The results show a possible correlation between glycated CD36 levels and alterations in glucose metabolism. However, these differences were not statistically significant, though this may be due to the limited number of subjects. A larger sample size will helps us to corroborate these results and determine if there is a relationship between mRNA and preDM or DM development.

In summary, our results have shown that hyperglycemic conditions increase glycated/non-glycated expression. This overexpression appears in prediabetic stages and can induce the early development of atherosclerosis in those with subdiabetic levels of hyperglycemia.
